# Translation, cross-cultural adaptation and validation of the Mandarin version of the BDDQ-AS for rhinoplasty patients

**DOI:** 10.1186/s40463-022-00557-x

**Published:** 2022-02-05

**Authors:** Wilson A. Wijaya, Yu Liu, Min Zhou, Yong Qing, Zhengyong Li

**Affiliations:** Department of Burn and Plastic Surgery, West China Hospital, Sichuan University, No.37 Guoxue Alley, Wuhou District, Chengdu City, Sichuan Province 61000 People’s Republic of China

**Keywords:** Body dysmorphic disorder, Rhinoplasty, Aesthetic surgery, Questionnaire, Mandarin, Cosmetic surgery

## Abstract

**Background:**

The BDDQ-AS (Body Dysmorphic Disorder Questionnaire—Aesthetic Surgery) is a simple and reliable patient-reported outcome measure. It can be used as a screening tool for body dysmorphic disorder (BDD) in patients undergoing aesthetic rhinoplasty. The aim of this study was to translate and culturally adapt the Mandarin version of the BDDQ-AS (M-BDDQ-AS) and evaluate its selected psychometric validation in patients after rhinoplasty.

**Method:**

According to international guidelines, the BDDQ-AS questionnaire was translated from English to Mandarin. Twenty Mandarin-speaking rhinoplasty patients were interviewed in order to evaluate the understandability and acceptability of the translation and produce a final version. It was then administered to 137 patients with a mean age of 38.75 ± 6.24 years. Psychometric validation were evaluated using reliability (internal consistency, test–retest reliability) and item-reponse theory (IRT) test.

**Result:**

High internal consistency of 0.823 was found using Cronbach’s α coefficient. Reliability of the M-BDDQ-AS resulted in Intraclass Correlation Coefficient (ICC) = 0.863. Besides, based on IRT analysis, the discrimination abilities of all the items were good (over 2.0), and their difficulty estimates were reasonable.

**Conclusion:**

The M-BDDQ-AS is a reliable and valid self-reported questionnaire that can be used in rhinoplasty patients. The very good psychometric validation of the M-BDDQ-AS indicates that it can be used in clinical practice and scientific research.

**Graphical Abstract:**

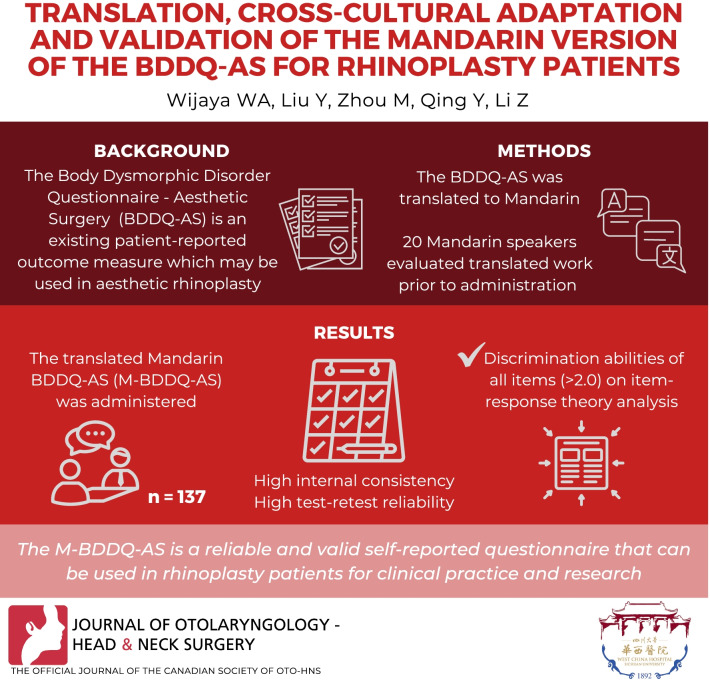

## Introduction

Body dysmororphic disorder (BDD) is a chronic psychiatric illness that is difficult to diagnose, causes significant anxiety, and is difficult to cure. The Diagnostic and Statistical Manual of Mental Disorders (DSM-5) defines BDD as preoccupation with one or more perceived defects or flaws in the physical appearance that are unobservable or appear minor to others [1]. The preoccupation induces clinically significant distress or impairment in sociological, occupational, or other areas of functioning [2]. Time-consuming, compulsive, repetitive behaviors or mental acts done excessively in response to preoccupation with appearance are the primary symptoms of BDD. The individual feels compelled to examine, improve, or conceal the perceived deficiency on a regular basis [3].

To improve their perceived blemish and alleviate these symptoms, people with BDD often seek cosmetic surgery. According to studies, between 71 and 76 percent of patients with BDD seek aesthetic therapy [4, 5]. This statistic is problematic because (1) BDD is underrecognized in aesthetic surgery practices and (2) studies suggest that patients with BDD need psychiatric treatment, not aesthetic surgery [4–8]. Several studies [3, 7, 8] show that aesthetic treatments rarely improve BDD, and patients with BDD who receive aesthetic treatments are usually dissatisfied with the outcome. Cosmetic treatment not only does not help the patient with BDD, but also exposes the surgeon to undue risk. Patients with BDD who undergo aesthetic treatment often take up the surgeon's time with frequent phone calls and requests for additional consultations and procedures. These patients also tend to file malpractice suits and may even become physically violent toward their surgeons [8–11]. These problems underscore the need for accurate identification of patients with BDD presenting for aesthetic procedures.

In recent years, numerous studies have shown that BDD is more common in patients seeking aesthetic treatment. To assess the extent and morbidity of BDD in these patients, many researchers have developed questionnaires to evaluate and manage this type of disease [12]. The BDDQ-AS (Body Dysmorphic Disorder Questionnaire—Aesthetic Surgery) is an easy-to-use, investigator-independent screening tool for BDD symptoms in people who are having aesthetic rhinoplasty [12]. This study showed that the sensitivity and specificity of the questionnaire were 89.6% and 81.4% for patients with at least moderate BDD symptoms. Patients with positive BDDQ-AS screening had significantly lower postoperative satisfaction with the final outcome [12].

To date, there is no available Mandarin version of the BDDQ-AS questionnaire to test its effectiveness for Mandarin-speaking rhinoplasty patients. The purpose of this study is to perform translation, cultural adaptation, and validation of the Mandarin version of the BDDQ-AS (M-BDDQ-AS).

## Methods

### BDDQ-AS

The Body Dysmorphic Disorder Questionnaire-Aesthetic Surgery (BDDQ-AS) was developed by Lekakis et al. [12] It is a validated seven-item brief questionnaire used as a screening tool for body dysmorphic disorder (BDD) in aesthetic rhinoplasty patients. It consists of three dichotomous questions with "yes/no" answers and four questions with five-point Likert scales indicating severity. Screening is considered positive for BDD if the patient confirmed on the BDDQ-AS that he or she worries (question 1 = yes) and worries about his or her appearance (question 2 = yes) and that these worries cause him or her at least moderate distress or impairment in various areas of daily life (question 3 or 4 or 5 or 6 ≥ 3 or question 7 = yes).

### Translation and cross-cultural adaptation

According to the guidelines, the translation and cross-cultural adaptation of the original English version of the BDDQ-AS into the Mandarin version was carried out. Two native Mandarin speakers with appropriate medical knowledge (Y.L and M.Z) independently translated the questionnaire into simplified Chinese and produced two translation versions. Two preliminary M-BDDQ-AS versions were independently translated back into English by two bilingual expert translators and a plastic surgeon who were blinded to the original English version of the questionnaire. Finally, an expert committee was formed by the two forward translators, two backward translators, a methodologist, and the researchers. The two preliminary M-BDDQ-AS versions and the two backward translated English versions were compared with the original English version to obtain a pre-final M-BDDQ-AS version. To obtain a final version of the questionnaire, a pilot test was conducted with two test groups. The first group consisted of 20 native Chinese-speaking patients selected from our outpatient clinics and determined face validity using a dichotomous scale (clear/unclear). These 20 patients were not included in the final validation. The second group consisted of three senior plastic surgeons in the burns and Plastic Reconstructive Surgery department of West China Hospital and three dermatologists in the dermatovenerology department of West China Hospital, who determined the content validity index using a four-point Likert scale (1 = not relevant to 4 = very relevant). The content validity index for the items (I-CVI) and the content validity index for the scale (S-CVI) were calculated according to a previously published equation. Six members of the expert committee analyzed the pilot test results, including feedback and recommendations from the two test groups that identified specific changes that were needed in the pre-final questionnaire. Their work resulted in the final M-BDDQ-AS.

### Study population

The study was conducted at the Burn and Plastic Reconstructive Surgery Department of West China Hospital of Sichuan University, a tertiary referral hospital in the People's Republic of China. One hundred and thirty-seven native Chinese patients seeking rhinoplasty were consecutively selected at the outpatient clinic. Exclusion criteria were inability to understand the questionnaire, severe physical deformities due to tumors or other diseases, and psychotic disorders.

### Psychometric validation

A total of 137 patients were included in the validation phase of the study. Two surveys were conducted by an online survey platform, WJX (https://www.wjx.cn/). For the first survey, each patient was asked to scan a QR code that contained the corresponding M-BDDQ-AS questionnaire, an informed consent form, and a description of the study. All 137 patients responded to the first survey (7 male, 130 female). The second survey took place 2 weeks after the first survey (test–retest). Of these, 71 patients responded to the second survey (5 male, 66 female).

Psychometric validation of the final M-BDDQ-AS, including reliability (internal consistency, test–retest reliability), and item response theory analysis were examined as follows:

(1) Internal consistency: the Cronbach's α coefficient was used to examine the internal consistency of the instrument's reliability, and values equal to or greater than 0.7 were considered consistent. The Cronbach's α coefficient was calculated along with a two-sided 95% confidence interval (CI).

(2) Test–retest reliability: the reproducibility of the M-BDDQ-AS was verified by Intraclass Correlation Coefficient (ICC) by assessing the reliability of the responses between the first and second surveys. The analysis is based on the resolution index, and a value equal to or greater than 0.6 indicates that the instrument had a good level of reproducibility.

(3) Item response theory (IRT): the discrimination and difficulty parameters of the questionnaire were defined. A discrimination parameter describes the sensitivity of the test to distinguish symptoms of different severity. A difficulty parameter refers to the point of median probability at which 50% of the respondents affirm the correct answer on the questionnaire.

### Ethical considerations

Prior to conducting the study, permission was obtained from Medical Ethics Committee the West China Hospital of Sichuan University.

### Statistical analysis

Descriptive statistics were used to assess baseline demographic variables and pooled responses. Psychometric validation of the M-BDDQ-AS questionnaire was determined to assess its performance. All *p*-values were considered statistically significant if they were less than 0.05.

All the above statistical analyses were performed using Stata Data Analysis and Statistical Software, version 14.0 (StataCorp LP).

## Results

A total of 137 patients completed the questionnaires, and the baseline demographic variables are shown in Table 1. The mean age of the patients was 38.75 ± 6.24 years, and 94.89% were women. Majority of the patients were non-smokers (91.24%) while the others were smokers. More than half of the patients were married (53.28%). In terms of educational level, 10.95% had a high school diploma, 11.68% had a graduate degree, 52.55% had a bachelor's degree, 19.71% had a master's degree, and 5.11% had a doctorate degree. Most patients came to our clinics for aesthetic surgery consultation (97.08%).

**Table 1:** Demographic characteristics of the participants (*n* = 137)

Patient characteristic	No. (%)
**Age,** mean(SD), y	38.75(6.24)
**Gender**	
Male	7(5.11)
Female	130(94.89)
**Smoking**	
Yes	12(8.76)
No	125(91.24)
**Marital status**	
Unknown	0(0)
Separated/ divorced	6(4.38)
Single	58(42.34)
Married	73(53.28)
**Education**	
High school	15(10.95)
Diploma degree	16(11.68)
Bachelor degree	72(52.55)
Master degree	27(19.71)
Doctoral degree	7(5.11)
**Visit type**	
Reconstructive sugery	4(2.92)
Aesthetic surgery	133(97.08)

We were able to translate, adapt, and validate the BDDQ-AS into Mandarin, resulting in the M-BDDQ-AS. This Mandarin version proved to be conceptually and psychometrically equivalent to the original English version (Table 2). This multi-stage process is important not only to achieve semantic equivalence, but also to ensure that the original content and concepts are respected and adapted to the objectives of the people involved in the instrument.

**Table 2:** Comparison between the original BDDQ-AS version and the adapted translated and back translation version

Item	BDDQ-AS original scale	BDDQ-AS adapted translated version (pre-final)	Back translation
Q1	Are you very worried about your appearance in any way? Yes / No	你会在各个方面都担心自己的外表不好看吗? 是 / 否	**Are you anxious about your appearance in any way?** Yes / No
Q2	Does these concerns preoccupy you? That is, do you think about it a lot and do you wish you could worry about it less? Yes / No	这些问题是否困扰着您? 如果是, 你是否会经常想起这些问题, 你是否希望你能减少对这些问题的担忧? 是 / 否	Does these concerns preoccupy you? That is, do you think about it a lot and do you wish you could worry about it less? Yes / No
Q3	Did these concerns cause you a lot of distress torment or pain? (Circle the best answer) 1 = No 2 = Mild, not too disturbing 3 = Moderate, disturbing but still manageable 4 = Severe, very disturbing 5 = Extreme disabling	这些困扰会给您带来很多痛苦吗?(选出最佳答案) 1 = 一点也不 2 = 轻微, 并不构成困扰 3 = 中等, 已造成困扰但是可以控制 4 = 严重, 非常困扰 5 = 极其困扰	Did these concerns cause you a lot of distress torment or pain? (Circle the best answer) 1 = No 2 = Mild, not too disturbing 3 = Moderate, disturbing but still manageable 4 = Severe, very disturbing 5 = Extreme disabling
Q4	Did these concerns cause you impaired in social, occupational or other important areas of functioning? (Circle the best answer) 1 = No 2 = Mild, not too disturbing 3 = Moderate, disturbing but still manageable 4 = Severe, very disturbing 5 = Extreme disabling	这些担忧是否会影响您的社交、职业或其他重要方面的功能?(选出最佳答案) 1 = 一点也不 2 = 轻微, 并不构成困扰 3 = 中等, 已造成困扰但是可以控制 4 = 严重, 非常困扰 5 = 极其困扰	Did these concerns cause you impaired in social, occupational or other important areas of functioning? (Circle the best answer) 1 = No 2 = Mild, not too disturbing 3 = Moderate, disturbing but still manageable 4 = Severe, very disturbing 5 = Extreme disabling
Q5	Did these concerns often significantly interfere with your social life? (Circle the best answer) 1 = No 2 = Mild, not too disturbing 3 = Moderate, disturbing but still manageable 4 = Severe, very disturbing 5 = Extreme disabling	这些担忧是否经常严重干扰您的社交生活?(选出最佳答案) 1 = 一点也不 2 = 轻微, 并不构成困扰 3 = 中等, 已造成困扰但是可以控制 4 = 严重, 非常困扰 5 = 极其困扰	Did these concerns often significantly interfere with your social life? (Circle the best answer) 1 = No 2 = Mild, not too disturbing 3 = Moderate, disturbing but still manageable 4 = Severe, very disturbing 5 = Extreme disabling
Q6	Did these concerns often significantly interfere with your schoolwork, job or ability to function in your role? (Circle the best answer) 1 = No 2 = Mild, not too disturbing 3 = Moderate, disturbing but still manageable 4 = Severe, very disturbing 5 = Extreme disabling	这些担忧是否经常严重影响你的学业、工作或履行职责的能力?(选出最佳答案) 1 = 一点也不 2 = 轻微, 并不构成困扰 3 = 中等, 已造成困扰但是可以控制 4 = 严重, 非常困扰 5 = 极其困扰	Did these concerns often significantly interfere with your schoolwork, job or ability to function in your role? (Circle the best answer) 1 = No 2 = Mild, not too disturbing 3 = Moderate, disturbing but still manageable 4 = Severe, very disturbing 5 = Extreme disabling
Q7	Are there things you avoid because of these concerns? Yes / No	你是否会因为这些担忧而避免去做一些事情? 是 / 否	Are there things you avoid because of these concerns? Yes / No

In the pilot testing phase and finalisation of the final M-BDDQ-AS questionnaire, content validity results showed that the M-BDDQ-AS had an S-CVI score of 0.95. All I-CVI scores ranged from 0.83 to 1.00, as described in Table 3. The final version of the M-BDDQ-AS is shown in Table 4.

**Table 3:** I-CVI and S-CVI scores for the M-BDDQ-AS

BDDQ-AS domain	Item	Expert 1	Expert 2	Expert 3	Expert 4	Expert 5	Expert 6	I-CVI
**Hybrid models(Q1, Q2, Q7)**	Q1	3	4	2	4	4	4	0.83
	Q2	4	4	4	4	4	4	1
	Q7	4	4	4	4	4	4	1
**Graded response sub-models(Q3-Q6)**	Q3	4	4	4	4	4	4	1
	Q4	4	3	4	4	4	3	0.83
	Q5	4	4	4	4	4	4	1
	Q6	4	4	4	4	4	4	1
	S-CVI	1	1	1	1	0.86	0.81	0.95

I-CVI, content validity index for items, S-CVI, content validity index for scale

**Table 4:**
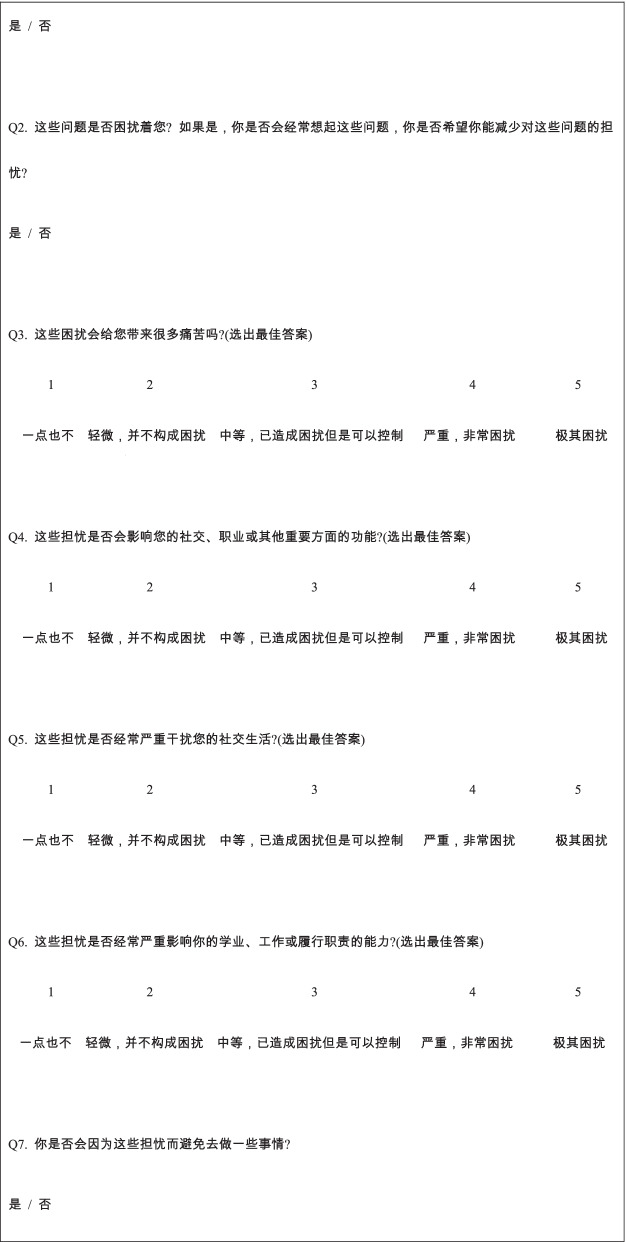
Final version of Mandarin BDDQ-AS (M-BDDQ-AS)

The M-BDDQ-AS questionnaire is a reliable instrument, all items of the M-BDDQ-AS showed very good internal consistency (all items Cronbach's α > 0.7, with the sum of Cronbach's α = 0.823) and excellent test–retest reliability (ICC = 0.863) (Table 5).

**Table 5:** Cronbach’s alpha results for internal consistency evaluation and Intraclass Correlation Coefficient results for test–retest reproducibility

M-BDDQ-AS	Cronbach’s α* (consistency) n = 137	ICC (95% CI)
Total	α = 0.823	0.863 (0.795–0.931)
Q1	α = 0.776	0.783 (0.715–0.851)
Q2	α = 0.802	0.828 (0.76–0.896)
Q3	α = 0.904	0.922 (0.854–0.99)
Q4	α = 0.866	0.878 (0.81–0.946)
Q5	α = 0.892	0.910 (0.842–0.978)
Q6	α = 0.854	0.876 (0.808–0.944)
Q7	α = 0.799	0.826 (0.758–0.894)

^*^The *p-values* for all the estimates < 0.05

Based on IRT analysis, the discrimination abilities of all items were good (> 2.0, p < 0.05), and their difficulty estimates were good (Table 6).

**Table 6:** Discrimination and difficulty abilities of M-BDDQ-AS

Items	Estimate	95% CI
Q1		
Discrimination	3.21	3.02–3.40
Difficulty	0.38	0.33–0.43
Q2		
Discrimination	2.95	2.60–3.30
Difficulty	0.26	0.24–0.29
Q3		
Discrimination	10.28	10.10–10.50
Difficulty		
≥ 2	0.58	0.54–0.62
≥ 3	0.93	0.86–1.01
≥ 4	1.57	1.46–1.69
5	1.98	1.83–2.13
Q4		
Discrimination	7.06	6.71–7.41
Difficulty		
≥ 2	0.36	0.21–0.51
≥ 3	0.93	0.73–1.13
≥ 4	1.69	1.49–1.89
5	2.13	1.93–2.33
Q5		
Discrimination	8.55	8.36–8.75
Difficulty		
≥ 2	0.25	0.05–0.44
≥ 3	0.88	0.68–1.08
≥ 4	1.24	1.04–1.44
5	1.84	1.65–2.04
Q6		
Discrimination	5.89	5.54–6.24
Difficulty		
≥ 2	0.78	0.54–1.02
≥ 3	1.53	1.29–1.78
≥ 4	1.98	1.74–2.22
5	2.76	2.45–3.07
Q7		
Discrimination	3.96	3.61–4.31
Difficulty	0.74	0.70–0.78

The *p-values* for all the estimates < 0.05

## Discussion

The BDDQ-AS was translated into Mandarin, culturally adapted, and reliability and validity tests were conducted. To ensure the accuracy of the cross-culturally adapted M-BDDQ-AS questionnaire, general guidelines for cross-cultural adaptation of instruments were followed [13–15]. The instrument was evaluated by health professionals who had worked with patients with body dysmorphic disorder and rhinoplasty.

The M-BDDQ-AS proved reliable and showed high internal consistency, which was also evident in the English version of the BDDQ-AS [12]. At the same time, the reliability and validity evaluation shows that this approach is both reliable and effective in the Chinese population and has a high degree of cultural adaptability. There was a significant positive correlation between the items of the M-BDDQ-AS. The methodology used in the translation process is also the guarantee of the validity of the content.

The limitations of this study include its small sample size and the fact that most of the patients were women. It is important to mention that the sample could have been more widely represented. Many other translation studies [16–18]; on the other hand, have performed a validation process with a similar or smaller number of participants. Furthermore, a small sample size would only reduce the likelihood of finding a significant association. We expect the correlations to be much stronger with a larger sample size because our findings demonstrate the reliability and validity of the M-BDDQ-AS, especially with the IRT. In other words, patients with more severe body dysmorphic symptoms performed well on the M-BDDQ-AS.

In this study, the M-BDDQ-AS was constructed first, and reliable results were obtained after rigorous testing and statistical analysis. This questionnaire can be promoted for use among Mandarin-speaking rhinoplasty patients and may provide a basis for future research in body dysmorphic disorder.

## Conclusion

This study demonstrated that the Mandarin version of the BDDQ-AS is a reliable and valid self-reported questionnaire that can be used to evaluate the functional and cosmetic outcomes of rhinoplasty patients. It is a valuable tool that can contribute to the screening of candidates with body dysmorphic disorder in Mandarin-speaking rhinoplasty patients.

## Data Availability

The datasets used and/or analysed during the current study are available from the corresponding author on reasonable request.
